# The impact of damage-associated molecules released from canine tumor cells on gene expression in macrophages

**DOI:** 10.1038/s41598-021-87979-1

**Published:** 2021-04-19

**Authors:** Shotaro Eto, Hideyuki Yanai, Sho Hangai, Daiki Kato, Ryohei Nishimura, Takayuki Nakagawa

**Affiliations:** 1 Laboratory of Veterinary Surgery, Graduate School of Agricultural and Life Sciences, The University of Tokyo, 1-1-1 Yayoi, Bunkyo-ku, Tokyo, 113-8657 Japan; 2 Department of Inflammology, Research Center for Advanced Science and Technology, The University of Tokyo, Komaba 4-6-1, Meguro-ku, Tokyo, 153-8505 Japan

**Keywords:** Chemokines, Cell death and immune response

## Abstract

Dying or damaged cells that are not completely eradicated by the immune system release their intracellular components in the extracellular space. Aberrant exposure of the damage-associated molecules to the immune system is often associated with inflammation and cancer pathogenesis. Thus, elucidating the role of damage-associated molecules in inducing sterile immune responses is crucial. In this study, we show that prostaglandin E2 (PGE2) is produced in the supernatants from several types of canine necrotic tumor cell lines. Inhibition of PGE2 production by indomethacin, a potent inhibitor of cyclooxygenase (COX) enzymes, induces the expression of tumor necrosis factor (*Tnf*) mRNA in the necrotic tumor cell supernatants. These results comply with the previous observations reported in mouse cell lines. Furthermore, comprehensive ribonucleic acid-sequencing (RNA-seq) analysis revealed that three categories of genes were induced by the damage-associated molecules: (i) a group of PGE2-inducible genes, (ii) genes that promote inflammation and are suppressed by PGE2, and (iii) a group of genes not suppressed by PGE2. Collectively, our findings reveal the hitherto unknown immune regulatory system by PGE2 and damage-associated molecules, which may have clinical implications in inflammation and cancer.

## Introduction

In the human body, ~ 10^5^ cells die every second by programmed cell death^1^. These dead cells are quickly sensed and eradicated by the macrophages and other phagocytes^2^. Despite the protective mechanisms intended for the removal of dead cells, several recent studies have shown that excessive cell death beyond the phagocytic elimination limits can result in trauma and sterile inflammation, which are closely associated with the pathogenesis of cancer and autoimmune diseases^3^. Therefore, understanding the precise mechanisms underlying the induction of sterile inflammation by the self-derived, damage-associated molecules is crucial.

Dying and damaged cells release their intracellular components into the extracellular space. The self-derived, damage-associated molecules (also known as damage-associated molecular patterns; DAMPs^3–6^), induce activation of immune responses through innate immune pattern recognition receptors (PRRs) such as the Toll-like receptors (TLRs)^3–6^. To date, various types of immune-activating damage-associated molecules have been identified, including high-mobility group box protein 1 (HMGB1), heat shock proteins (HSPs), S100 proteins (calcium-binding cytosolic proteins), interleukin (IL)-1A, IL-33, nucleic acids (NAs), adenosine triphosphate (ATP), and uric acids^3–6^. Some of these are recognized by the TLRs and reportedly induce sterile inflammation^4^.

Although several damage-associated molecules have been identified, we unexpectedly found that the supernatants from necrotic dead cells did not induce the tumor necrosis factor-α (TNF-α), a potent pro-inflammatory cytokine induced by the TLR activation in peritoneal macrophages^7^. We also found that the supernatant contains a substantial amount of prostaglandin E2 (PGE2), which is synthesized by the cyclooxygenase (COX) enzymes, COX-1 and COX-2. PGE2 released from the dying or dead cells exerts immunosuppressive effects on macrophages via its receptors, EP2/EP4^8,9^. Indeed, *Tnf* mRNA induction by lipopolysaccharide (LPS), a TLR4 agonist, was suppressed by treatment with the necrotic supernatant. Furthermore, we also found that treatment of supernatants from necrotic tumor cells with indomethacin, an inhibitor of the COX enzymes, reduced the production of PGE2 and enhanced the expression of *Tnf* mRNA in macrophages^7^. Therefore, both immunoactive and immunosuppressive molecules released from dying or dead necrotic tumor cells, tune the resulting sterile inflammation.

Although immune responses induced by self-derived damage-associated molecules have been studied, the detail mechanisms underlying the regulation of sterile inflammation by the self-derived damage-associated molecules remain unclear. In addition, most studies have focused on human and mouse cells, while cells from other species have not been studied. Recently, dogs have been highlighted as a promising model for human diseases such as cancer and genetic disease, as many naturally occurring diseases in dogs closely resemble those in humans^10–12^. Dogs as a model organism provide an ideal solution to the gap between laboratory rodents and humans for research on translational medicine, but research on damage-associated molecules using canine cells is limited.

In this study, by employing a series of canine tumor cell lines, we showed that necrotic supernatants from all the cell lines did not induce *Tnf* mRNA expression in either canine or mouse macrophage cell lines. We also confirmed that necrotic supernatants from the canine tumor cells containing PGE2 and supernatants from indomethacin-treated cells induced *Tnf* mRNA expression in mouse macrophages. Furthermore, we performed comprehensive ribonucleic acid-sequencing (RNA-seq) to analyze the gene expression profiles in cells treated with necrotic tumor cell supernatants with or without indomethacin treatment. These data are believed to reveal information on the previously unknown regulatory mechanisms of innate immune responses by self-derived damage-associated molecules.

## Results

### Necrotic supernatants from most canine tumor cells did not induce *Tnf* mRNA expression in macrophages

**Figure 1:**
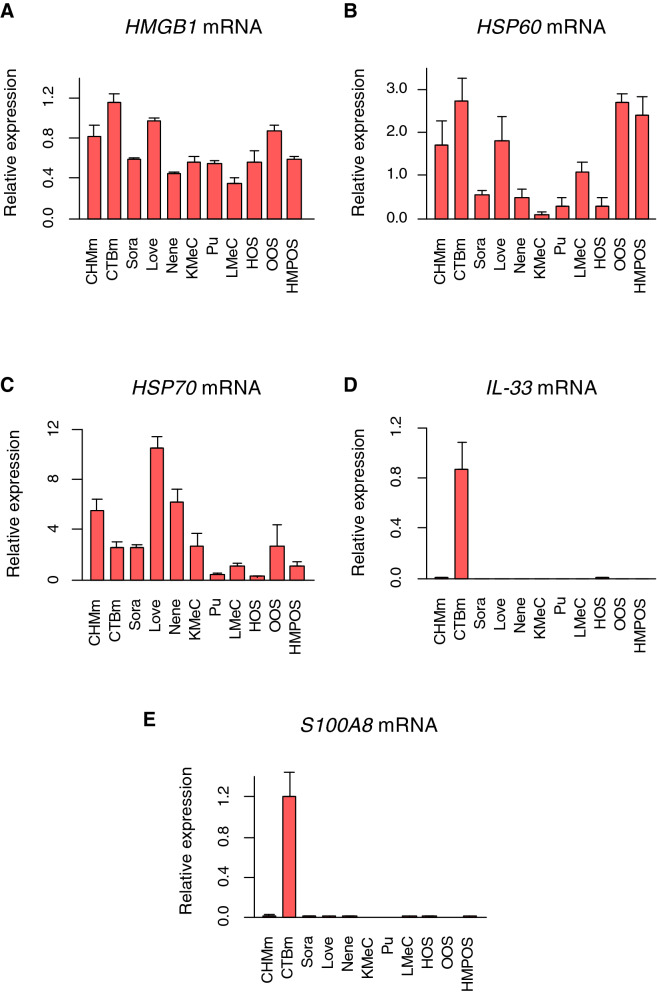
Gene expression of damage-associated molecules (*HMGB1*, *HSP60*, *HSP70*, *IL33*, *S100A8*) in 11 canine tumor cell lines. (**A**–**E**) Total RNA was extracted from cell lines in the logarithmic growth phase. *HMGB1* (**A**), *HSP60* (**B**), *HSP70* (**C**), *IL33* (**D**), and *S100A8* (**E**) mRNA expression levels were examined by RT-qPCR analysis. Data are presented as mean ± SD.

We first analyzed the mRNA expression levels of damage-associated molecules (*HMGB1*, *HSP60*, *HSP70*, *S100A8*, *IL-1A*, *IL-33*) in 11 canine tumor cell lines (Table 1). The 11 canine tumor cell lines comprised of two breast cancer (CHMm and CTBm), three urothelial cell carcinoma (Sora, Love, Nene), three malignant melanoma (KMeC, Pu, LMeC), and three osteosarcoma (HOS, OOS, HMPOS) cell lines. These four different types of canine tumors reportedly possess similarities with human tumors in terms of clinical behavior and molecular mechanisms^13–16^. Reverse transcription-quantitative polymerase chain reaction (RT-qPCR) analysis revealed that all canine tumor cells express several damage-associated molecules at various levels (Fig. 1). *Il1a* mRNA expression was not detected in these cancer cell lines. We also analyzed the mRNA expression of damaged-associated molecules in canine urothelial carcinoma tissues and adjacent normal tissues. The expression levels of damage-associated molecules in cancer tissues are not significantly different from those in normal tissues (Supplementary Fig.S1), indicating that damage-associated molecules are not highly expressed in cancer tissues but also in normal tissues.

**Table 1:** Summary of 11 canine tumor cell lines used in this study.

Tissue of origin	Cell lines	References
Mammary gland carcinoma	CHMm	^33^
	CTBm	^33^
Urothelial cell carcinoma	Sora	^34^
	Love	^34^
	Nene	Originally established
Malignant Melanoma	KMeC	^35^
	Pu	^36^
	LMeC	^35^
Osteosarcoma	HOS	^37^
	OOS	^37^
	HMPOS	^38,39^

**Figure 2:**
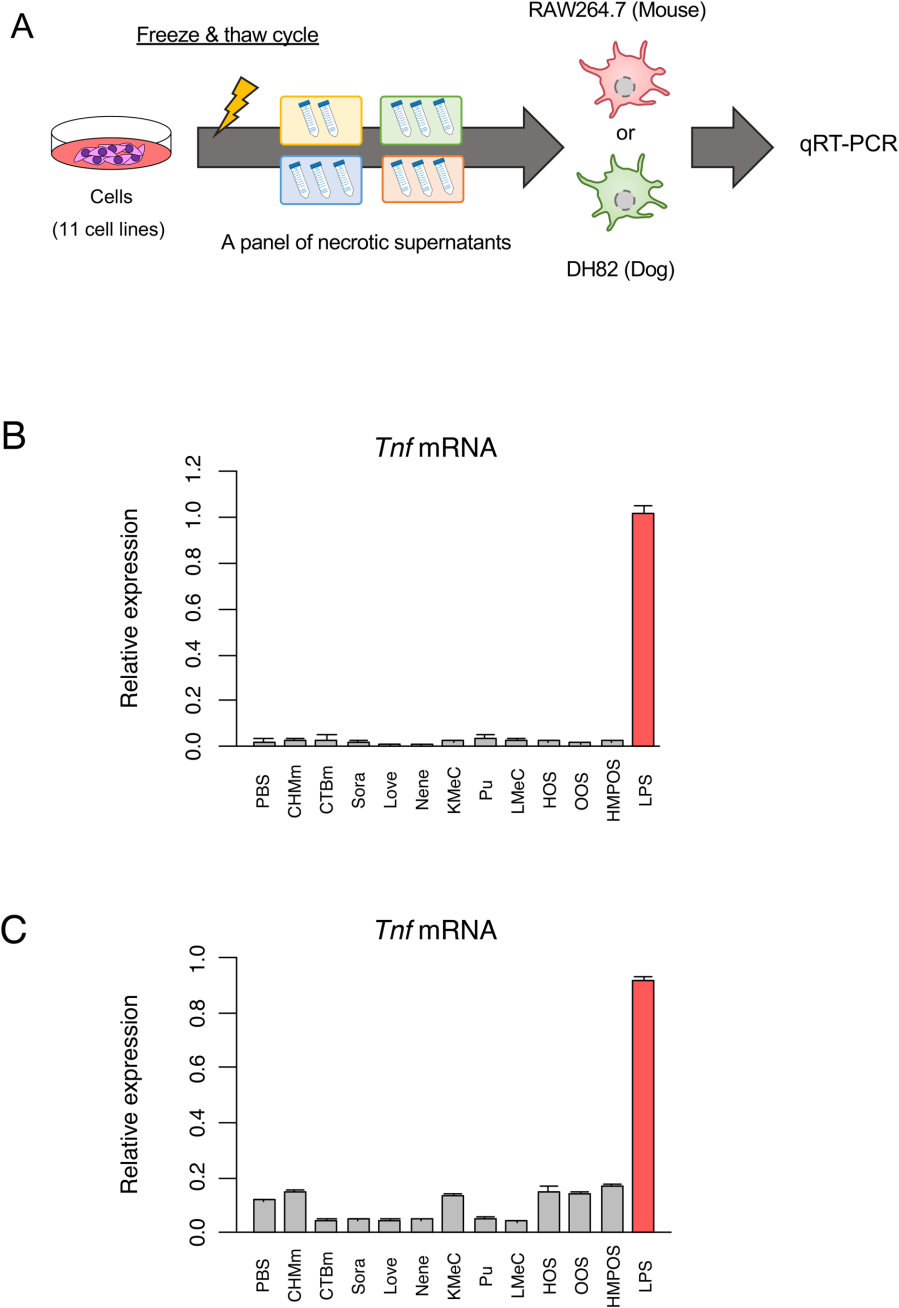
Lack of immunostimulatory activities of canine tumor cell-derived necrotic supernatants. (**A**) Schematic illustration of the preparation of necrotic tumor cell supernatants and RT-qPCR analysis for *Tnf* mRNA expression in macrophage cell lines. In each tumor cell line, necrosis was induced by freeze–thaw cycles. (**B,C**) Murine macrophages (RAW264.7) (**B**) and canine macrophages (DH82) (**C**) were exposed to the necrotic supernatant (the volume of the supernatant is equivalent to 5 × 10^6^ cells) for 2 h. *Tnf* mRNA expression was examined by RT-qPCR analysis. Data are presented as mean ± SD. Samples that showed more than two-fold induction compared to PBS samples are indicated in red bars.

We next prepared necrotic supernatants from 11 canine tumor cell lines by freeze–thaw cycles to examine whether the necrotic canine tumor cell-derived damage-associated molecules induce an inflammatory response. A murine macrophage cell line (RAW264.7) and a canine histiocytic sarcoma cell line (DH82) with macrophage-like phenotypes^17^ were treated with these necrotic canine tumor cell supernatants and then analyzed for *Tnf* mRNA expression (Fig. 2A). Although the representative damage-associated molecules were expressed in all canine cancer cell lines (Fig. 1), we did not observe the expression of *Tnf* mRNA expression in both RAW264.7 and DH82 cells, when treated with the necrotic tumor cell supernatants of all canine tumor cell lines (Fig. 2B,C). These data are consistent with our previous results showing that supernatants derived from necrotic murine cell lines do not induce *Tnf* mRNA expression^7^.

### PGE2 produced by dying and dead canine tumor cells suppresses innate immune activation

**Figure 3:**
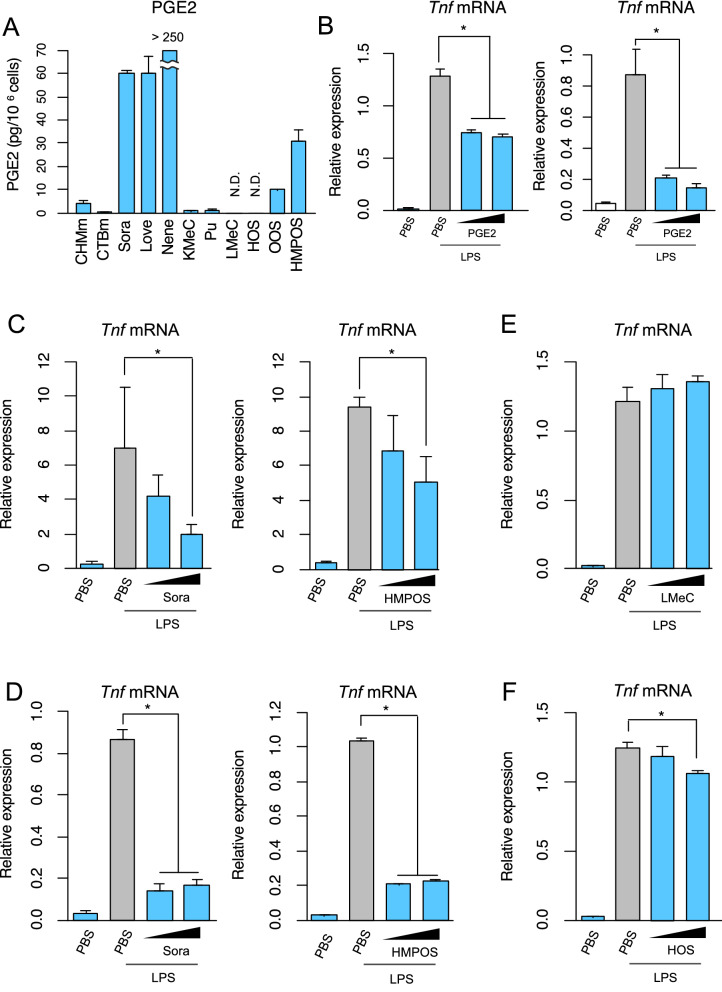
Suppression of LPS-induced *Tnf* mRNA expression by dying cells-derived necrotic supernatant or PGE2 treatment. (**A**) Production of PGE2 in necrotic supernatants derived from canine tumor cell lines. PGE2 in necrotic tumor cell supernatants was measured by ELISA and normalized to cell number. (**B**) RAW264.7 cells (left panel) and DH82 cells (right panel) were left untreated or stimulated with LPS (10 ng/ml) for 2 h in the presence of PGE2 (50 nM or 500 nM) or absence (PBS). *Tnf* mRNA expression levels were determined by RT-qPCR analysis. (**C,D**) RAW264.7 cells (**C**) and DH82 cells (**D**) were stimulated with LPS (10 ng/ml) for 2 h and co-cultured with an increasing volume of necrotic supernatants from Sora cells (left panels) and HMPOS (right panels). The volume of the supernatant is equivalent to 5 × 10^5^ or 5 × 10^6^ cells. Expression of *Tnf* mRNA was then measured by RT-qPCR analysis. (**E,F**) RAW264.7 cells were stimulated with LPS for 2 h in combination with or without the same volumes of necrotic supernatants from LMeC cells (**E**) and HOS cells (**F**). The induction levels for *Tnf* mRNA were determined by RT-qPCR. Data are presented as mean ± SD.*, p < 0.05 compared with the indicated samples.

We next sought to examine PGE2 production in 11 necrotic canine tumor cell supernatants, as PGE2 produced by necrotic murine tumor cells functions as a suppressor for the activation of innate immune responses by dead tumor cells^7^. Consistent with our previous report, PGE2 was significantly detected in 9 of 11 necrotic canine tumor cell supernatants (Fig. 3A). Based on these data, we next examined whether PGE2-containing necrotic canine tumor cell supernatants impede the activation of innate immune responses in RAW264.7 and DH82 cells. PGE2 treatment of RAW264.7 and DH82 cells suppressed *Tnf* mRNA expression induction by LPS stimulation in the macrophages (Fig. 3B), suggesting that PGE2 from canine tumor dead cells functions as an immunosuppressor. To examine the immunosuppressive activity of PGE2 in the necrotic canine tumor cell supernatants, we selected Sora and HMPOS cells because they showed relatively high PGE2 production in the supernatants (Fig. 3A). We stimulated the macrophages with LPS in combination with the necrotic canine tumor cell supernatants, and then examined *Tnf* mRNA expression. As shown in Fig. 3C,D, the PGE2-containing supernatants massively suppressed LPS-induced *Tnf* mRNA expression in both RAW264.7 and DH82 cells. The inhibition of LPS-induced TNF-α production by PGE2 was also observed at the protein levels by ELISA (Supplementary Fig. S2). In contrast, there was little, if any, suppressive activity in LMeC- or HOS-derived necrotic canine tumor cell supernatant (Fig. 3E,F), which does not contain PGE2, suggesting that PGE2 in the necrotic canine tumor cell supernatants plays an important role in *Tnf* mRNA suppression.

**Figure 4:**
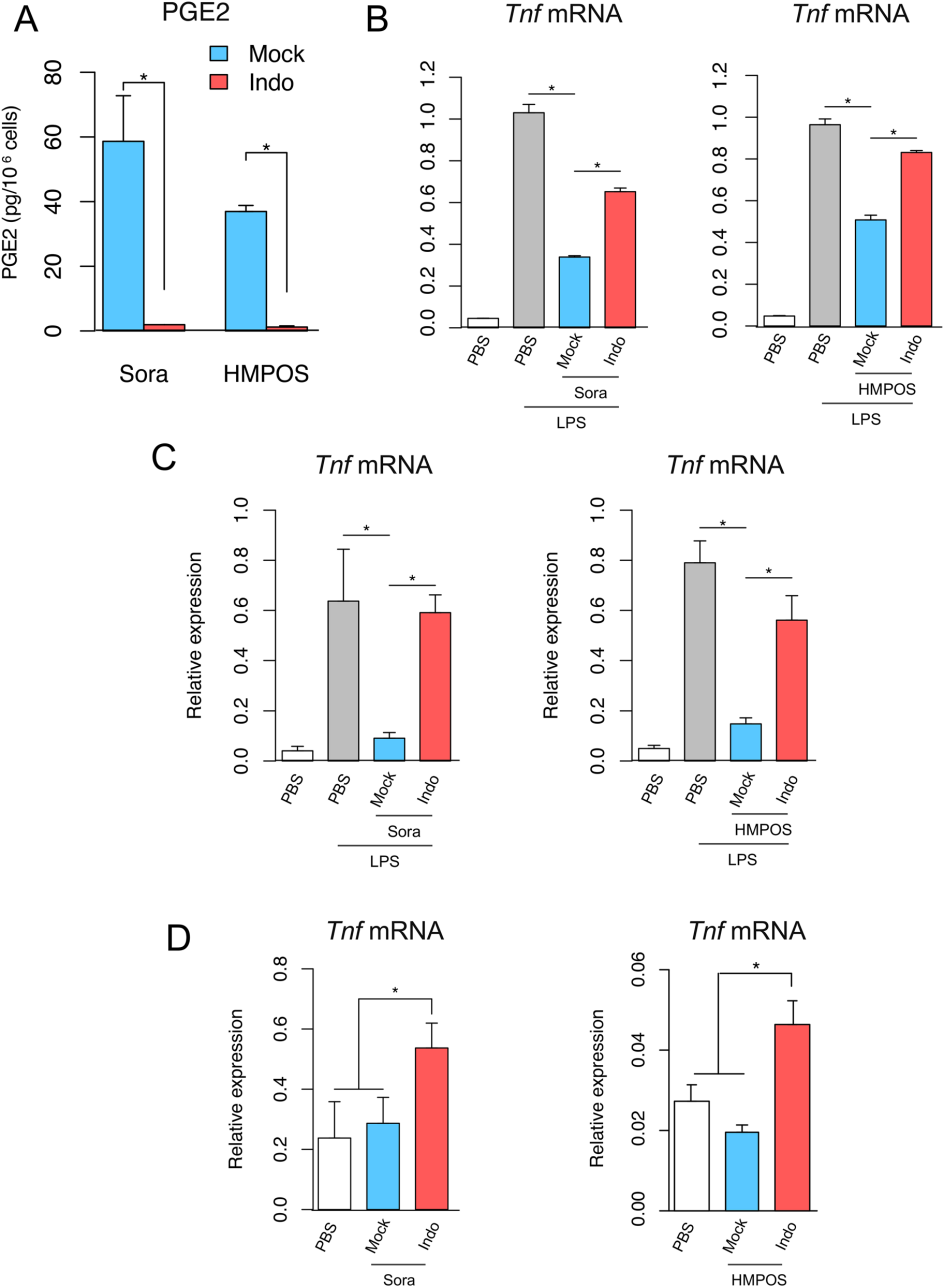
Suppression of PGE2 production and enhancement of immunostimulatory activity of necrotic canine tumor cell supernatants. (**A**) Production of PGE2 in necrotic supernatant from DMSO (Mock)- and indomethacin (Indo)-treated tumor cells. PGE2 levels were determined by ELISA and normalized to cell number. (**B,C**) RAW264.7 cells (**B**) and DH82 cells (**C**) were stimulated with LPS (10 ng/ml) for 2 h in combination with necrotic supernatants (5 × 10^6^ cells) from Sora cells (left panels) and HMPOS cells (right panels). The induction levels for *Tnf* mRNA were then determined by RT-qPCR analysis. (**D**) RAW264.7 cells were treated for 2 h with necrotic supernatants from Mock-or Indo-treated Sora (left panel) and HMPOS (right panel) cells (5 × 10^6^ cells). *Tnf* mRNA expression levels were examined by RT-qPCR analysis. Data are presented as mean ± SD. *p < 0.05 compared with the indicated samples.

Next, we examined whether necrotic canine tumor cell self-derived damage-associated molecules activate the innate immune responses in the absence of PGE2. We pretreated Sora and HMPOS cells with indomethacin, a potent inhibitor of the COX enzymes, and then prepared a necrotic supernatant of the cells. As expected, PGE2 production in the supernatant was markedly reduced by treatment with indomethacin (Fig. 4A). Notably, indomethacin-treated necrotic supernatants from canine tumor cell did not show any suppressive effect on LPS-induced *Tnf* expression in RAW264.7 and DH82 cells (Fig. 4B,C). We then treated LPS-unstimulated RAW264.7 cells with PGE2-reduced supernatant, which interestingly upregulated *Tnf* mRNA expression (Fig. 4D). TNF-α production in RAW264.7 cells was also observed (Supplementary Fig. S3). Collectively, these data indicate that dying and dead canine tumor cells release PGE2 and inhibit innate immune activation by damage-associated molecules.

We further examined how PGE2 released from necrotic cells suppresses LPS-induced *Tnf* mRNA expression in macrophage cell lines. We first used specific inhibitors targeting four different PGE2 receptors (EP1–EP4)^8,9^. The suppression of LPS-induced *Tnf* mRNA expression by necrotic cell supernatants was attenuated by EP2 and EP4 inhibitors, and the suppression was further significantly reduced by the combination of both inhibitors (Supplementary Fig. S4). These results indicated that PGE2 released from dying cells exerts its suppressive effect through the EP2 and EP4 receptors signaling. To investigate further the mechanism of immunosuppressive effect by PGE2-EP2/EP4 axis, we examined the activation of NF-κB, a critical transcription factor for TNF-α, by NF-κB luciferase reporter assay. Notably, the addition of necrotic cell supernatants did not inhibit the activation of NF-κB by LPS stimulation (Supplementary Fig. S5), suggesting that PGE2 in the supernatant suppresses induction of *Tnf* mRNA in an NF-kB-independent manner (See also “Discussion”).

### Gene expression profiles in macrophages treated with necrotic canine tumor cell supernatants in the presence and absence of PGE2

**Figure 5:**
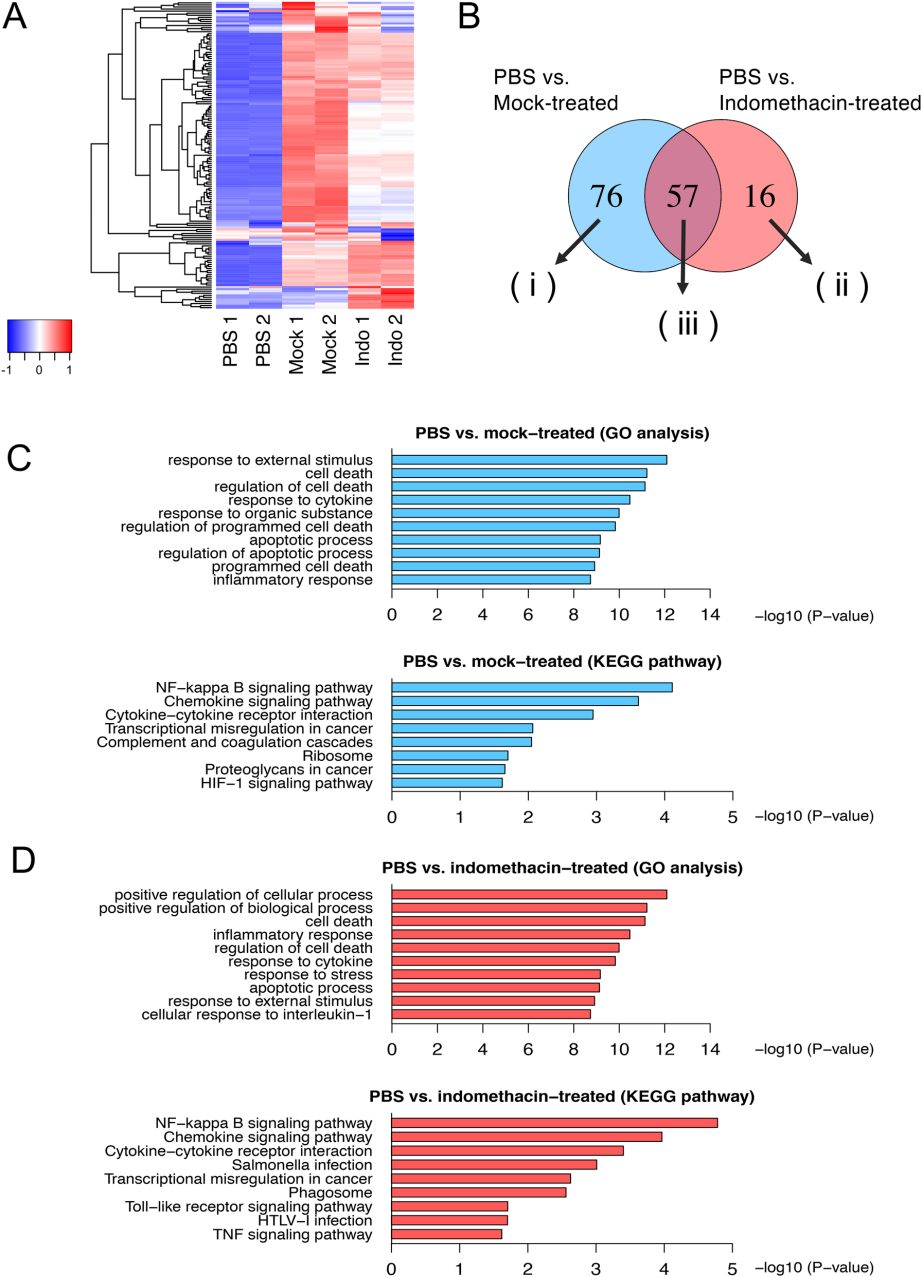
Gene expression analysis of RAW264.7 cells treated with necrotic supernatants by comprehensive RNA-seq analysis. RAW264.7 cells were treated for 4 h with necrotic supernatant from Sora cells pretreated with indomethacin (Indo) or DMSO (Mock). Total RNA was extracted and subjected to RNA-seq analysis. Differentially expressed genes (DEGs) were identified using FDR < 0.05 as the statistical criterion for filtering. (**A**) Heat map of the different gene expression patterns in each sample. (**B**) Venn diagrams showing commonly and differentially regulated genes by each necrotic supernatant. Group (i) contains 76 genes induced only by necrotic supernatant from Mock. Group (ii) represents 16 genes induced only by necrotic supernatant from Indo. Group (iii) includes 57 genes commonly induced by necrotic supernatants from Mock and Indo. (**C,D**) Biological processes and signaling pathways affected by necrotic supernatants from Mock (**C**) and Indo (**D**) Sora cells. GO analysis (upper panels) and KEGG pathway enrichment analysis (lower panels) were employed.

The induction of *Tnf* mRNA expression and TNF-α production in RAW264.7 cells by PGE2-reduced supernatants (Fig. 4D and Supplementary Fig. S3) prompted us to investigate which genes are induced by the damage-associated molecules by comprehensive gene expression analysis. We performed RNA-seq analysis with the total RNA extracted from the RAW264.7 cells treated with the necrotic supernatant from canine tumor cells subjected to indomethacin treatment. As shown in Fig. 5A,B, differential expression analysis identified 73 upregulated differentially expressed genes (DEGs) in the RAW264.7 cells treated with necrotic supernatant prepared from the Sora cells in the presence of indomethacin (Indo), compared to untreated cells (PBS). We also found 133 DEGs in the RAW264.7 cells treated with the necrotic supernatant derived from Sora cells in the absence of indomethacin (Mock). All DEGs and their fold changes are listed in Supplementary Table S1.

To gain further insights into the upregulated DEGs, we performed Gene Ontology (GO) analysis to examine the functional changes between PBS vs. Mock and between PBS vs. Indo. The analysis identified 164 (PBS vs. Mock) and 107 (PBS vs. Indo) GO terms with p-value < 0.01 and false discovery rate (FDR) < 0.01. The top 10 terms in each condition were dominated by terms involved in the activation of immune responses, such as response to external stimulus, inflammatory response, and response to cytokines (Fig. 5C,D). We also performed a Kyoto encyclopedia of genes and genomes (KEGG) pathway enrichment analysis and found 8 (PBS vs. Mock) and 9 (PBS vs. Indo) signal pathways with p-values < 0.05. Notably, the nuclear factor-kB (NF-kB) signaling was the most upregulated pathway under the stimulation by each necrotic supernatant from canine tumor cells (Fig. 5C,D).

**Figure 6:**
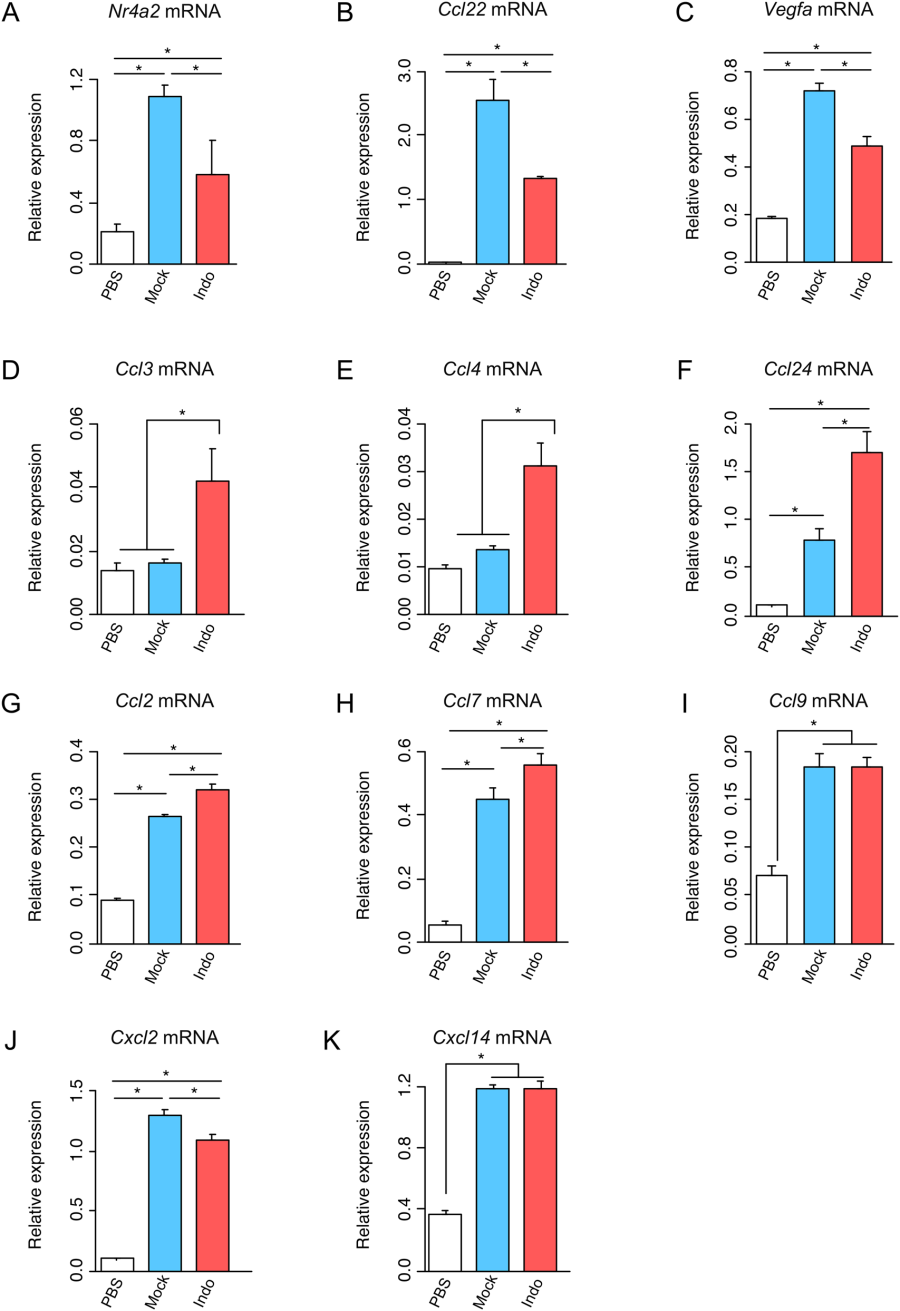
Validation of representative DEGs derived from RNA-seq analysis by RT-qPCR. (**A**–**K**) RAW264.7 cells were treated for 4 h with the necrotic supernatant from PBS or Indo. Gene expression of group (i) (*Nr4a2* (**A**), *Ccl22* (**B**), and *Vegfa* (**C**)), group (ii) (*Ccl3* (**D**), *Ccl4* (**E**), and *Ccl24* (**F**)), and group (iii) (*Ccl2* (**G**), *Ccl7* (**H**), *Ccl9* (**I**), *Cxcl2* (**J**), and *Cxcl14* (**K**)) was examined by RT-qPCR analysis. Data are presented as mean ± SD. *p < 0.05 compared with the indicated samples.

**Figure 7:**
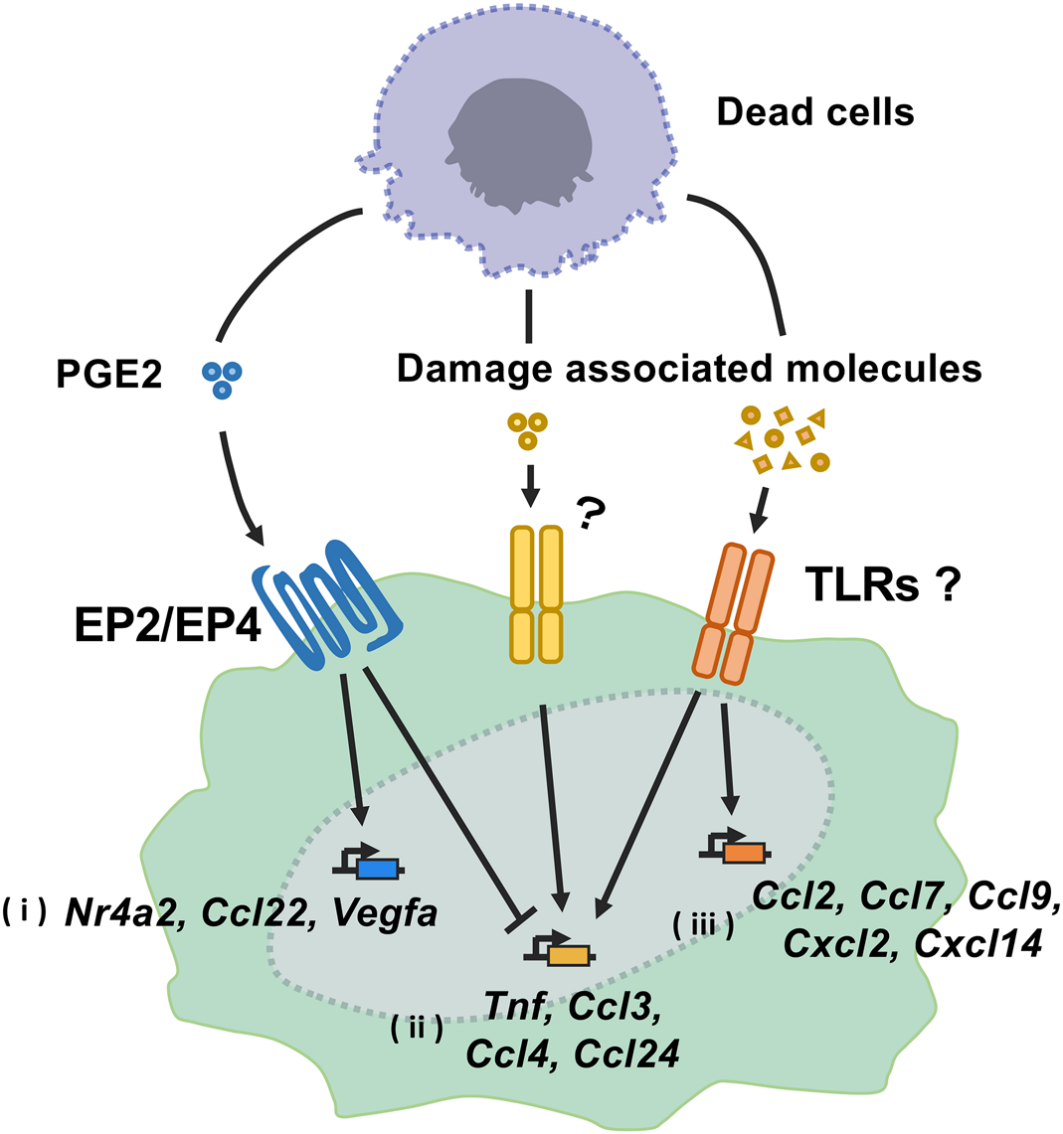
Schematic diagram of the interaction of PGE2 and damage-related molecules in the induction of genes regulating the immune response in macrophages. PGE2 released from dead cells induces immunosuppressive molecules such as *Nr4a2*, *Ccl22,* and *Vegfa* via EP1-4, PGE2 receptors (i). Damage-associated molecules induce a variety of pro-inflammatory chemokines, probably via TLRs. Some of these genes (*Tnf*, *Ccl3*, *Ccl4* and *Ccl24*) are suppressed by PGE2 (ii) and others (*Ccl2*, *Ccl7*, *Ccl9*. *Cxcl2* and *Cxcl14*) are not (iii). Since there are no reports on the relationship between *Ccl24* mRNA and TLR signaling, new damage-related molecules may be involved in the induction of *Ccl24* mRNA.

The Venn diagram shows that 76 DEGs (group i) were selectively upregulated by the necrotic supernatant from Mock (Fig. 5B), indicating that PGE2 affects expression of several DEGs. These DEGs include *Ccl22* and *Nr4a2* (Supplementary Table S1), which are reported to be induced by PGE2^18,19^. Sixteen DEGs (group ii) were specifically upregulated by PGE2-reduced necrotic supernatant (Fig. 5B). These DEGs included *Tnf* and pro-inflammatory chemokines such as *Ccl3*, *Ccl4*, and *Ccl24* (Supplementary Table S1). Fifty-seven DEGs (group iii) including *Ccl2*, *Ccl7*, *Ccl9*, *Cxcl2*, and *Cxcl14* chemokines were upregulated in both Indo and PBS (Fig. 5B), indicating that the damage-associated molecules induce these DEGs in a PGE2-independent manner. These DEGs Expression profiles of the genes were confirmed by measuring the relative mRNA levels through RT-qPCR analysis (Fig. 6). Taken together, these data revealed hitherto unknown complex immune regulatory mechanisms by immune-activating damage-associated molecules and PGE2 (Fig. 7).

## Discussion

In the recent years, numerous studies have reported that innate immune activation by the self-derived damage-associated molecules obtained from dead cells promotes inflammation, which exacerbates pathogenesis of cancer, autoimmune diseases, and chronic inflammation^3–6^. Although damage-associated molecules have gained attention for their pro-inflammatory activity^3–6^, it remains unclear whether and how the damage-associated molecules regulate the inflammatory innate immune responses. To further characterize the activation of innate immune responses by the damage-associated molecules, in this study, we examined innate immune activation by several types of damage-associated molecules obtained from the canine tumor cell lines.

Consistent with our previous findings^7^, PGE2 was indeed produced in the necrotic supernatants from majority of the canine tumor cell lines (9/11) (Fig. 3A). In particular, the necrotic supernatants from 3 cell lines of urothelial carcinoma (Sora, Love, and Nene) contained large amounts of PGE2 (Fig. 3A). This observation may reflect our previous result that the COX genes are highly expressed in these cell lines^20,21^.

Furthermore, despite the fact that LMeC-derived supernatants without PGE2 production did not suppress LPS-induced *Tnf* mRNA expression, the HOS-derived supernatants also without PGE2 production, showed relatively weak innate immune suppression at high concentrations (Fig. 3E,F). These results suggest the presence of previously unidentified new immunosuppressive damage-associated molecule(s) in the supernatant. Identification and characterization of the immunosuppressive molecule(s) released from dying cells need further clarification.

Based on our data and previous reports^8,9^, PGE2 released from dying cells exerted its immunosuppressive effects mainly through EP2 and EP4 receptors (Supplementary Fig.S4). Although the mechanism of suppression of inflammation induction by the PGE2-EP2/4 axis is not well understood, we observed PGE2 treatment did not affect the transcriptional activation of NF-κB (Supplementary Fig. S5). In this context, we found that 76 DEGs (group i) including *Nr4a2, Ccl22 and Vegfa* were induced specifically by PGE2-containing necrotic supernatant (Figs. 5B, 6A–C, Supplementary Table S1). It is plausible that PGE2 in the supernatant induces the expression of the genes^18,19,22,23^. Since Nr4a2 is known to suppress induction of *Tnf* mRNA expression by LPS stimulation in monocytes/macrophages in an NF-κB-independent manner^24^, the suppression of *Tnf* mRNA induction may be mediated by Nr4a2. In addition, CCL22 promotes the migration of regulatory T cells (Tregs) through the CCR4 receptor^25,26^, and VEGF-A in addition to its angiogenic effects exerts immunosuppressive effects leading to the accumulation of Tregs and myeloid-derived suppressor cells in the tumor microenvironment^27^. Therefore, PGE2 released from the dying and dead cells may suppress inflammation and promote wound healing through the induction of these immunosuppressive mediators.

RNA-seq analysis also revealed that there are two groups of DEGs induced by damage-associated molecules (Fig. 5B). One group consisted of 16 DEGs (group ii), including *Tnf*, *Ccl3*, *Ccl4*, and *Ccl24*, whose expressions are suppressed by PGE2 (Figs. 5B, 6D–F, Supplementary Table S1). Since PGE2 is known to suppress the expression of *Ccl3* and *Ccl4* induced by TLR2/TLR4^28^, damage-associated molecules may activate the TLR signaling pathway. In contrast, *Ccl24* mRNA expression is mainly induced by the T helper cell type 2 (Th2) cytokines, such as IL-4 and IL-13^29^, but its association with TLR signaling has not been reported. Therefore, it is expected that a new damage-associated molecule may be involved in the induction of *Ccl24* mRNA. Another group contained 57 DEGs (group iii), including *Ccl2*, *Ccl7*, *Ccl9*, *Cxcl2*, and *Cxcl14*. The expression of these DEGs was unaffected by indomethacin treatment (Figs. 5B, 6G–K). This indicates that the induction of these mRNA was not repressed by PGE2. Furthermore, KEGG pathway analysis suggested that the NF-kB signaling pathway was activated by damage-associated molecules (Fig. 5C,D). Activation of the NF-kB signaling pathway has been observed in a variety of inflammatory diseases and may contribute to the exacerbation of disease pathology^30^. It remains to be clarified which damage-associated molecules activate the NF-kB signaling pathway.

In the GO analysis, several terms related to cell death were included in the top of the list in addition to those related to immune response (Fig. 5C,D). Originally, TLR signaling, extracellular HMGB1 and histones induce apoptosis in macrophages^31^; however, in the present study, necrosis supernatants enhanced the expression of the genes involved in anti-apoptosis, such as *Bcl2a1d*, *Bcl2a1b*, and *Bcl2l1*. This may be involved in the activation of NF-kB signaling, reported to suppress apoptosis downstream of TLRs^32^, but it is unknown how damage-related molecules promote cell survival in macrophages.

In summary, this study reveals a hitherto unknown mechanism for the regulation of innate immune responses by the damage-associated molecules. The identification of the damage-associated molecules involved in the regulation of innate immune responses may be critical for understanding their role in the immune regulatory processes and clinical pathogenesis of inflammatory diseases.

## Methods

### Reagents and cell culture

LPS (O55:B4), SC51089 (EP1 inhibitor), TG4-155(EP2 inhibitor), L-798106 (EP3 inhibitor) and ONO-AE3-208 (EP4 inhibitor) was purchased from Sigma-Aldrich. PGE2 and indomethacin were purchased from Cayman Chemical. These reagents were reconstituted in dimethyl sulfoxide (DMSO) or phosphate buffered saline (PBS) and stored at − 20 °C or − 80 °C. Two canine mammary gland tumor cell lines (CHMm and CTBm), 3 canine urothelial cell carcinoma cell lines (Sora, Love, Nene), 3 canine malignant melanoma cell lines (KMeC, Pu, LMeC), and 3 canine osteosarcoma cell lines (HOS, OOS, HMPOS) used in this study were established in our laboratory^33–39^. We confirmed no mycoplasma contamination in any of these cell lines using the TaKaRa PCR Mycoplasma Detection Set (TaKaRa Bio). Each canine tumor cell line was maintained in Roswell Park Memorial Institute (RPMI) 1640 Medium (RPMI-1640) supplemented with 10% heat-inactivated fetal bovine serum (FBS) and 5 mg/L gentamicin (Sigma-Aldrich). RAW264.7 cells were maintained as described previously^40^. DH82 cells were kindly provided by Dr. Uchida (Laboratory of Veterinary Pathology, The University of Tokyo) and was cultured in Dulbecco’s Modified Eagle’s medium (DMEM) supplemented with 10% heat-inactivated FBS and 5 mg/L gentamicin. All cell lines were cultured at 37 °C in a humidified atmosphere with 5% carbon dioxide (CO_2_).

### Clinical canine samples

Surgically resected canine urothelial carcinoma tissue samples and adjacent normal tissues from the same case (n = 4) were obtained from the archival collection of the University of Tokyo Veterinary Medical Center (samples collected in 2017–2018). The client of each hospital provided informed consent for the use of these samples for this study.

### Preparation of necrotic tumor cell supernatants

Induction of necrosis was performed by a freeze–thaw method as described previously^41,42^. For the preparation of a PGE2-depleted necrotic supernatant, cells were treated with indomethacin (10 μM) for 24 h and subjected to freeze–thaw cycles in the presence of indomethacin (10 μM).

### Enzyme-linked immunosorbent assay (ELISA)

PGE2 concentration in the necrotic cell supernatant was determined using PGE2 ELISA Kit (Cayman Chemical). TNF-α production levels were examined using mouse TNF-α DuoSet ELISA (R&D Systems) according to the manufacturer’s instructions.

### Reporter assay

RAW 264.7 cells (5 × 10^6^) were seeded on 10 cm Petri dishes and transiently co-transfected with X-tremeGENE 9 DNA transfection reagent (Roche Life Science) and 5 μg firefly luciferase reporter plasmid for NF-κB^43^ (Stratagene) on the following day. After 24 h, cells were re-seeded on 24-well plates and stimulated with LPS (10 ng/mL) in the presence or absence of necrotic cell supernatants. After 2 h of incubation, cells were harvested with lysis buffer and luciferase activity was measured using PicaGene Luminescence Kit (Toyo BeNet Co., Ltd.).

### RT-qPCR analysis

Total RNA from tissues or cells was extracted using RNAiso (TaKaRa Bio) or NucleoSpin RNA II (Macherey–Nagel) and reverse-transcribed with PrimeScript RT Master Mix (TaKaRa Bio). RT-qPCR was performed on a LightCycler 480 using the SYBR Green PCR Master Mix (Roche Life Science), and values were normalized to the expression of *Gapdh* mRNA. Primer sequences are listed in Supplementary Table S2.

### RNA-sequencing (RNA-seq) analysis

Total RNA was extracted from RAW264.7 cells treated with PBS or treated with necrotic supernatants obtained from tumor cell lines with (Indo) or without (Mock) indomethacin treatment using NucleoSpin RNA II (Macherey–Nagel). RNA-seq (150 bp paired-end) analysis was performed by GENEWIZ using DNBSEQ-G400 sequencer, generating a minimum of 4.5 million read pairs for each sample. In order to remove technical sequences, including adapters, polymerase chain reaction (PCR) primers, or fragments thereof, and quality of bases lower than 20, pass filter data of fastq format were processed by Cutadapt (V1.9.1). Data were aligned to reference genome via software Hisat2 (v2.0.1). For gene expression analysis, FPKM (Fragments per kilo bases per million reads) was calculated based on the number of reads in HTSeq (v0.6.1). Differential gene expression analysis was performed using EdgeR^44,45^ using an FDR < 0.05 as the cutoff criterion. GO and KEGG pathway enrichment analysis were performed using the Database for Annotation, Visualization, and Integrated Discovery (DAVID, https://david.ncifcrf.gov/, version 6.8)^46,47^. For GO analysis, p-value < 0.01 and FDR < 0.01 were used as cutoff criteria. A p-value < 0.05 was used for the KEGG functional analysis.

### Data availability

The data obtained during the current study will be available from the corresponding author upon reasonable request. RNA-seq data were deposited in the E-GEAD-394.

### Statistical analysis

Statistical analysis was performed using R software (https://www.R-project.org/). Data are expressed as mean ± SD. The p-values were determined by Tukey–Kramer analysis and paired T test, and the difference was considered to be statistically significant at p < 0.05.

## Supplementary Information

Supplementary Information 1.

Supplementary Information 2.
