# Supplementary material for: The impact of damage-associated molecules released from canine tumor cells on gene expression in macrophages
Source: Sci Rep. 2021 Apr 19;11:8525. doi: 10.1038/s41598-021-87979-1 (PMC8055655; doi:10.1038/s41598-021-87979-1)
Supplement: Supplementary file 1 — Supplementary Information 1. [file 41598_2021_87979_MOESM1_ESM.pdf]

Supplementary Table S1. List of genes upregulated in RAW264.7 cells by Sora cell-derived necrotic supernatants identified by RNA-seq analysis. Genes were listed in each of three categories; (i) genes induced only by Mock, (ii) genes induced only by Indo, and (iii) genes induced by both Mock and Indo (Figure 5B).

Group (i)

Gene ID	Gene Symbol	PBS vs. mock-treated (log2FC)	PBS vs. indomethacin-treated (log2FC)
ENSMUSG00000037742	Eef1a1	0.073455156	-0.056990975
ENSMUSG00000030144	Clec4d	0.958666248	0.419378027
ENSMUSG00000064370	mt-Cytb	0.167952377	0.030536071
ENSMUSG00000030142	Clec4e	1.252651472	0.616547519
ENSMUSG00000064341	mt-Nd1	0.151558416	0.041485262
ENSMUSG00000006932	Ctnnb1	0.940638858	0.595546097
ENSMUSG00000031278	Acsl4	1.331324222	0.871205531
ENSMUSG00000032399	Rpl4	0.082257998	0.035840286
ENSMUSG00000001131	Timp1	2.350078649	1.2581717
ENSMUSG00000031779	Ccl22	5.121726691	3.325316626
ENSMUSG00000023951	Vegfa	2.530067691	1.638457625
ENSMUSG00000032122	Slc37a2	1.460769552	0.967071176
ENSMUSG00000063457	Rps15	0.103783873	-0.051887139
ENSMUSG00000028965	Tnfrsf9	4.904659862	3.160836415
ENSMUSG00000027907	S100a11	0.749747583	0.4052797
ENSMUSG00000062353	Gm15772	0.131200766	0.005865003
ENSMUSG00000020399	Havcr2	1.305351541	0.595934195
ENSMUSG00000059195	Gm12715	0.921541586	0.760601197
ENSMUSG00000103847	Gm20056	1.442616337	1.064722024
ENSMUSG00000087249	Gm16062	2.117465268	1.204631413
ENSMUSG00000074743	Thbd	3.32305812	2.188864171
ENSMUSG00000063234	Gpr84	0.834851254	0.699442172
ENSMUSG00000026271	Gpr35	3.078173266	2.298583307
ENSMUSG00000023068	Nus1	1.066117445	0.656463401
ENSMUSG00000027171	Prrg4	2.164216106	1.424220347
ENSMUSG00000036478	Btg1	1.433319648	1.040444184
ENSMUSG00000022272	Myo10	1.516826957	1.13063237
ENSMUSG00000023944	Hsp90ab1	0.144606684	0.017751219
ENSMUSG00000003970	Rpl8	0.099068537	0.014066453
ENSMUSG00000030695	Aldoa	0.118812444	-0.007067119
ENSMUSG00000042228	Lyn	0.996895536	0.587044081
ENSMUSG00000063889	Crem	2.729853918	1.722765965
ENSMUSG00000033161	Atp1a1	0.635713633	0.554445934
ENSMUSG00000078812	Eif5a	0.120209198	0.018127231
ENSMUSG00000027447	Cst3	0.529027228	0.364511239
ENSMUSG00000084349	Rpl3-ps1	0.087809583	-0.044289858
ENSMUSG00000080845	Gm9115	2.73222196	1.849712906
ENSMUSG00000027435	Cd93	2.317730863	1.39612861
ENSMUSG00000026177	Slc11a1	1.029879852	0.838363847
ENSMUSG00000045092	S1pr1	1.419471612	0.896964091
ENSMUSG00000047180	Neurl3	1.414038416	0.998764859
ENSMUSG00000040747	Cd53	0.665176524	0.556008069

ENSMUSG00000027611	Procr	1.953815148	1.170656685
ENSMUSG00000023048	Prr13	1.376838725	1.061336636
ENSMUSG00000004040	Stat3	1.724365371	1.115749167
ENSMUSG00000063229	Ldha	0.127982199	0.01602801
ENSMUSG00000038172	Ttc39b	1.457725796	1.068571808
ENSMUSG00000032518	Rpsa	0.104373376	-0.002434019
ENSMUSG00000064367	mt-Nd5	0.167031855	-0.004094724
ENSMUSG00000026832	Cytip	4.376170294	2.437927834
ENSMUSG00000058755	Osm	2.519724988	1.871268102
ENSMUSG00000007891	Ctsd	0.098319214	0.17754683
ENSMUSG00000029373	Pf4	0.970953185	0.737351299
ENSMUSG00000032802	Srxn1	0.881797149	0.400781651
ENSMUSG00000026875	Traf1	2.735186268	1.917129118
ENSMUSG00000025804	Ccr1	1.987986396	1.467448927
ENSMUSG00000025393	Atp5b	0.087548012	-0.070680594
ENSMUSG00000039361	Picalm	1.117870987	0.628041915
ENSMUSG00000026074	Map4k4	1.25954563	0.748952484
ENSMUSG00000074272	Ceacam1	2.17647367	1.764522507
ENSMUSG00000029135	Fosl2	1.841619652	0.986712424
ENSMUSG00000075122	Cd80	1.517982942	0.995986728
ENSMUSG00000060600	Eno3	1.568197727	1.488154974
ENSMUSG00000012848	Rps5	0.100148769	0.03436128
ENSMUSG00000018293	Pfn1	0.139485736	0.213143912
ENSMUSG00000021822	Plau	0.110532983	0.482796866
ENSMUSG00000015837	Sqstm1	0.706265941	0.599601554
ENSMUSG00000021998	Lcp1	0.074328121	-0.055012108
ENSMUSG00000026826	Nr4a2	2.864709818	1.325257539
ENSMUSG00000022303	Dcstamp	2.351441906	1.898613807
ENSMUSG00000029338	Antxr2	1.671273506	0.804189556
ENSMUSG00000028081	Rps3a1	0.166977379	-0.007240539
ENSMUSG00000002847	Pla1a	1.459007366	1.146581683
ENSMUSG00000063193	Cd300lb	0.805343829	0.695530338
ENSMUSG00000052485	Tmem171	1.912737964	1.607194443
ENSMUSG00000029762	Akr1b8	0.828454136	0.648100328

Group (ii)

Gene ID	Gene Symbol	PBS vs. mock-treated (log2FC)	PBS vs. indomethacin-treated (log2FC)
ENSMUSG00000000982	Ccl3	0.561249555	1.66794366
ENSMUSG00000018930	Ccl4	0.536892948	1.615535864
ENSMUSG00000063696	Gm8730	0.213241806	0.125165971
ENSMUSG00000100801	Gm15459	0.275539195	0.156036426
ENSMUSG00000021701	Plk2	0.052424085	2.074078056
ENSMUSG00000034994	Eef2	0.080951903	0.146143992
ENSMUSG00000046721	Rpl14-ps1	0.002177233	0.144377679
ENSMUSG00000058126	Tpm3-rs7	0.101482357	0.098059611
ENSMUSG00000004207	Psap	-0.009436238	0.119149399

ENSMUSG00000032575	Manf	0.652624609	0.733758444
ENSMUSG00000074622	Mafb	0.603933208	1.096557105
ENSMUSG00000004814	Ccl24	2.15735478	2.650518424
ENSMUSG00000021939	Ctsb	-0.038637986	0.117815134
ENSMUSG00000007659	Bcl2l1	0.539236925	0.32389974
ENSMUSG00000052837	Junb	0.785883071	0.937218217
ENSMUSG00000020368	Canx	0.158815875	0.097358242

Group (iii)

Gene ID	Gene Symbol	PBS vs. mock-treated (log2FC)	PBS vs. indomethacin-treated (log2FC)
ENSMUSG00000011179	Odc1	3.05881215	2.3149456
ENSMUSG00000107092	Gm7993	3.017051147	2.377521101
ENSMUSG00000051439	Cd14	1.347044113	0.732310638
ENSMUSG00000037820	Tgm2	3.708834616	2.710879306
ENSMUSG00000035385	Ccl2	5.121726691	2.356119145
ENSMUSG00000003541	Ier3	2.776691191	2.192921849
ENSMUSG00000049130	C5ar1	2.008912723	1.912076028
ENSMUSG00000001473	Tubb6	1.632160851	1.637016834
ENSMUSG00000021508	Cxcl14	2.341519862	2.173816024
ENSMUSG00000024737	Slc15a3	2.589180502	1.896450925
ENSMUSG00000034765	Dusp5	4.770992924	3.876104633
ENSMUSG00000045763	Basp1	2.331762121	1.923205233
ENSMUSG00000099974	Bcl2a1d	1.695516401	1.487693464
ENSMUSG00000026981	Il1rn	3.167585971	2.685374607
ENSMUSG00000019122	Ccl9	1.283610133	1.138576889
ENSMUSG00000047945	Marcksl1	1.243511349	1.085666224
ENSMUSG00000015243	Abca1	3.060252776	1.797875861
ENSMUSG00000058427	Cxcl2	4.218954119	3.787933251
ENSMUSG00000002944	Cd36	1.113298465	0.991684905
ENSMUSG00000015396	Cd83	3.232193575	2.078169057
ENSMUSG00000005087	Cd44	1.425011148	1.215653465
ENSMUSG00000062825	Actg1	0.928718164	0.768989739
ENSMUSG00000089929	Bcl2a1b	1.723188328	1.50289549
ENSMUSG00000027333	Smox	2.067083574	1.637786615
ENSMUSG00000082718	Gm14928	1.201870106	0.677230302
ENSMUSG00000039208	Metrnl	1.670835201	1.693986547
ENSMUSG00000020483	Dynll2	1.175100502	0.912755264
ENSMUSG00000091952	Gm17709	2.601502197	1.848596212
ENSMUSG00000000318	Clec10a	3.419162481	2.713614501
ENSMUSG00000020077	Srgn	1.115148867	0.952263704
ENSMUSG00000026864	Hspa5	0.612663164	0.629562507
ENSMUSG00000023272	Creld2	0.919009353	0.869640344
ENSMUSG00000003948	Mmd	1.61593652	1.722531586
ENSMUSG00000005514	Por	1.253279701	0.984260189
ENSMUSG00000035373	Ccl7	2.290155501	2.382613875
ENSMUSG00000043421	Hilpda	2.127481325	1.762935587

ENSMUSG00000002233	Rhoc	1.171995053	1.398263449
ENSMUSG00000042349	Ikbke	2.072003684	2.090514591
ENSMUSG00000024912	Fosl1	2.304033161	2.085640257
ENSMUSG00000020205	Phlda1	3.057795472	3.195510193
ENSMUSG00000056201	Cfl1	0.08053918	0.083432003
ENSMUSG00000032115	Hyou1	0.817093773	0.835077477
ENSMUSG00000046223	Plaur	1.545520402	1.816199644
ENSMUSG00000030162	Olr1	2.646142449	2.262963228
ENSMUSG00000000184	Ccnd2	1.788170285	1.968498106
ENSMUSG00000034993	Vat1	0.974193823	1.112254776
ENSMUSG00000042842	Serpinb6b	2.930049378	2.623557445
ENSMUSG00000032487	Ptgs2	1.530477523	1.73574706
ENSMUSG00000031504	Rab20	1.912718537	1.98299805
ENSMUSG00000049775	Tmsb4x	0.15654966	0.13083115
ENSMUSG00000029570	Lfng	1.279305637	1.29175264
ENSMUSG00000102037	Bcl2a1a	1.93753274	1.746317695
ENSMUSG00000037411	Serpine1	1.658784576	1.78659173
ENSMUSG00000039910	Cited2	0.660363775	0.85771615
ENSMUSG00000016319	Slc25a5	0.270680951	0.402240545
ENSMUSG00000022895	Ets2	0.960187246	1.050525518
ENSMUSG00000025492	Ifitm3	0.193440473	0.192422701

Supplementary Table S2. Primer sequences used in this study.

Mouse

Gene	Froward primer	Reverse primer	Accession No.
Gapdh	CTCATGACCACAGTCCATGC	CACATTGGGGGTAGGAACAC	NM_001289726.1
Ccl2	ACCTGGATCGGAACCAAATG	AGTGCTTGAGGTGGTTGTGG	NM_011333.3
Ccl3	AGCCAGGTGTCATTTTCCTG	ACTCTCAGGCATTCAGTTCCAG	NM_011337.2
Ccl4	TTCAGATTTCCTGCCCCTCTTC	CAGAGAAACAGCAATGGTGGAC	NM_013652.2
Ccl7	GACAAAGAAGGGCATGGAAGTC	GGCTTTGGAGTTGGGGTTTTC	NM_013654.3
Ccl9	GGTTAGCATTCCGTTGGCTAC	TGCCTGTGGTGAAAACCAAC	NM_011338.2
Ccl22	ATGGTGCCAATGTGGAAGAC	TCAAAACAACGCCAGGCTTG	NM_009137.2
Ccl24	CTGTGACCATCCCCTCATCT	CTTATGGCCCTTCTTGGTGA	NM_019577.5
Cxcl2	AAGTCATAGCCACTCTCAAGGGCG	TTGGTTCTTCCGTTGAGGGACAGC	NM_009140.2
Cxcl14	TGCGAGGAGAAGATGGTTATCG	GTTCCAGGCATTGTACCACTTG	NM_019568.2
Nr4a2	GCATACAGGTCCAACCCAGT	AATGCAGGAGAAGGCAGAAA	NM_013613.2
Tnf	TCATACCAGGAGAAAGTCAACCTC	GTATATGGGCTCATACCAGGGTTT	NM_013693.3
Vegfa	AGCAGAAGTCCCATGAAGTGA	ATGTCCACCAGGGTCTCAAT	NM_001025250.3

Dog

Gene	Froward primer	Reverse primer	Accession No.
Gapdh	TGACACCCACTCTTCCACCTTC	CGGTTGCTGTAGCCAAATTCA	NM_001003142.2
HMGB1	AAGTGAGAGCCAGACGGG	TCCTTTGCCCATGTTTAATTATTTTC	NM_001002937.2
HSP60	TGTCTTGGCACGCTCTATTG	TTGTCACGGGTTTAGACTGC	XM_025442140.2
HSP70	AAGTACAAAGCCGAGGACGAG	TCATGTTGAAGGCGTAGGACTC	NM_001003067.2
IL1A	ATGCCTGAGACACCCAAAAC	TTCGTTCCTGTGTGGCAATG	NM_001003157.2
IL33	ATGATGTTGATGGCCAGACG	TGTTCCTCGTTGTTGGCATG	NM_001003180.1
S100A8	TCCTCTGTCAACTCTGTTTCGG	TTTATGGCACTCTCCAGTTCCG	NM_001146144.1
Tnf	CATGTTGTAGCAAACCCCGAAG	ATGAGGTACAACCCATCTGACG	NM_001003244.4
