# Supplementary material for: The impact of damage-associated molecules released from canine tumor cells on gene expression in macrophages
Source: Sci Rep. 2021 Apr 19;11:8525. doi: 10.1038/s41598-021-87979-1 (PMC8055655; doi:10.1038/s41598-021-87979-1)
Supplement: Supplementary file 2 — Supplementary Information 2. [file 41598_2021_87979_MOESM2_ESM.pdf]

The impact of damage-associated molecules released from canine tumor cells on gene expression in macrophages

Shotaro Eto[1,2], Hideyuki Yanai[2], Sho Hangai[2], Daiki Kato[1], Ryohei Nishimura[1], Takayuki Nakagawa[1]*

[1] *Laboratory of Veterinary Surgery, Graduate School of Agricultural and Life Sciences, The University of Tokyo, 1-1-1 Yayoi, Bunkyo-ku, Tokyo 113-8657, Japan*

[2] *Department of Inflammology, Research Center for Advanced Science and Technology, The University of Tokyo, Komaba 4-6-1, Meguro-ku, Tokyo 153-8505, Japan*

* Corresponding author@ anakaga@g.ecc.u-tokyo.ac.jp

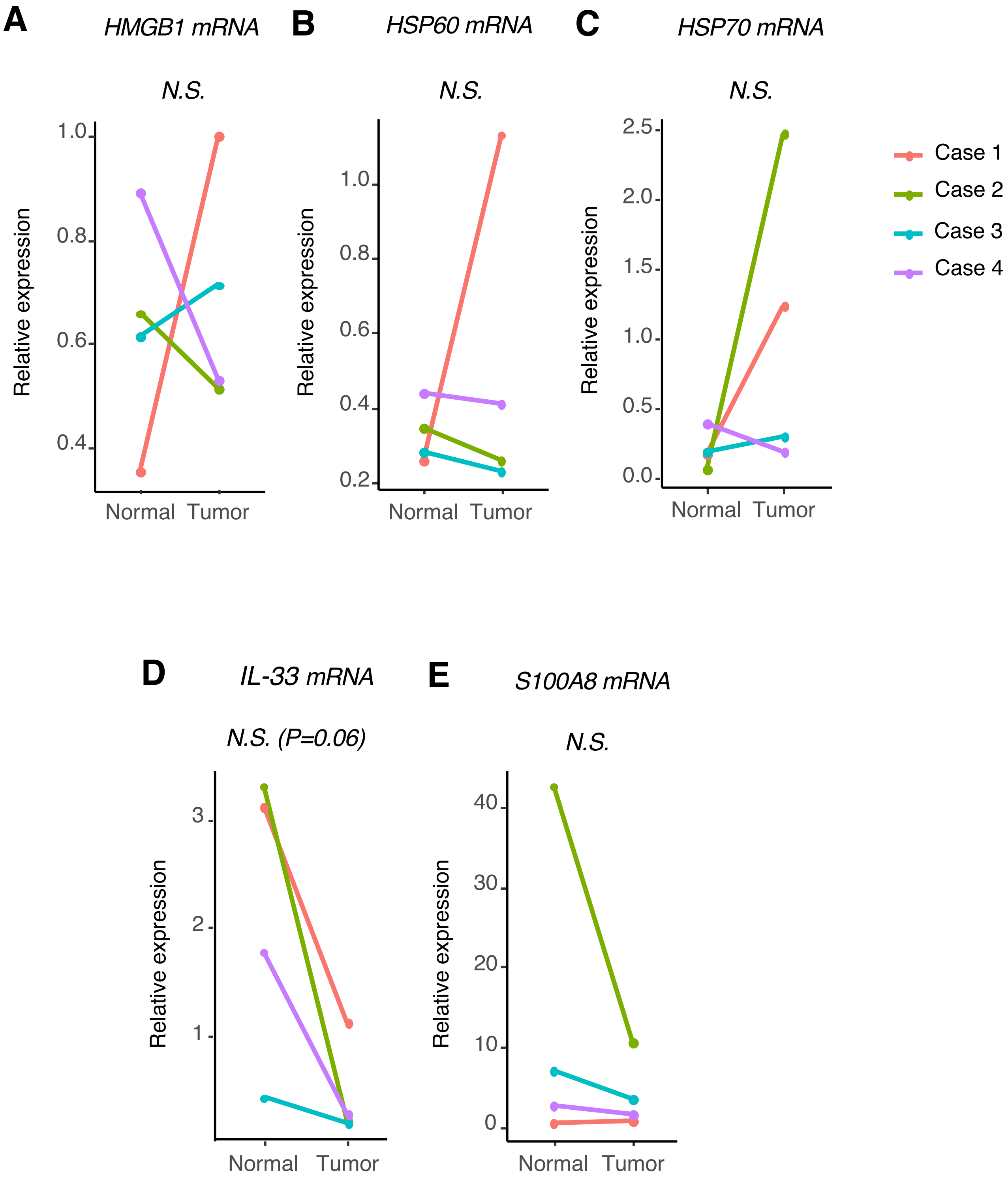

Supplementary Fig.S1

Expression levels of representative damaged associated molecules (*HMGB1*, *HSP60*, *HSP70*, *IL33*, *S100A8*) in tumor tissues and adjacent normal tissues in the same dogs with urothelial carcinoma (n=4). **(A - E)** Total RNA was extracted from tumor and normal tissues. *HMGB1* **(A)**, *HSP60* **(B)**, *HSP70* **(C)**, *IL33* **(D)**, and *S100A8* **(E)** mRNA expression levels were examined by RT-qPCR analysis. N.S.: not significant.

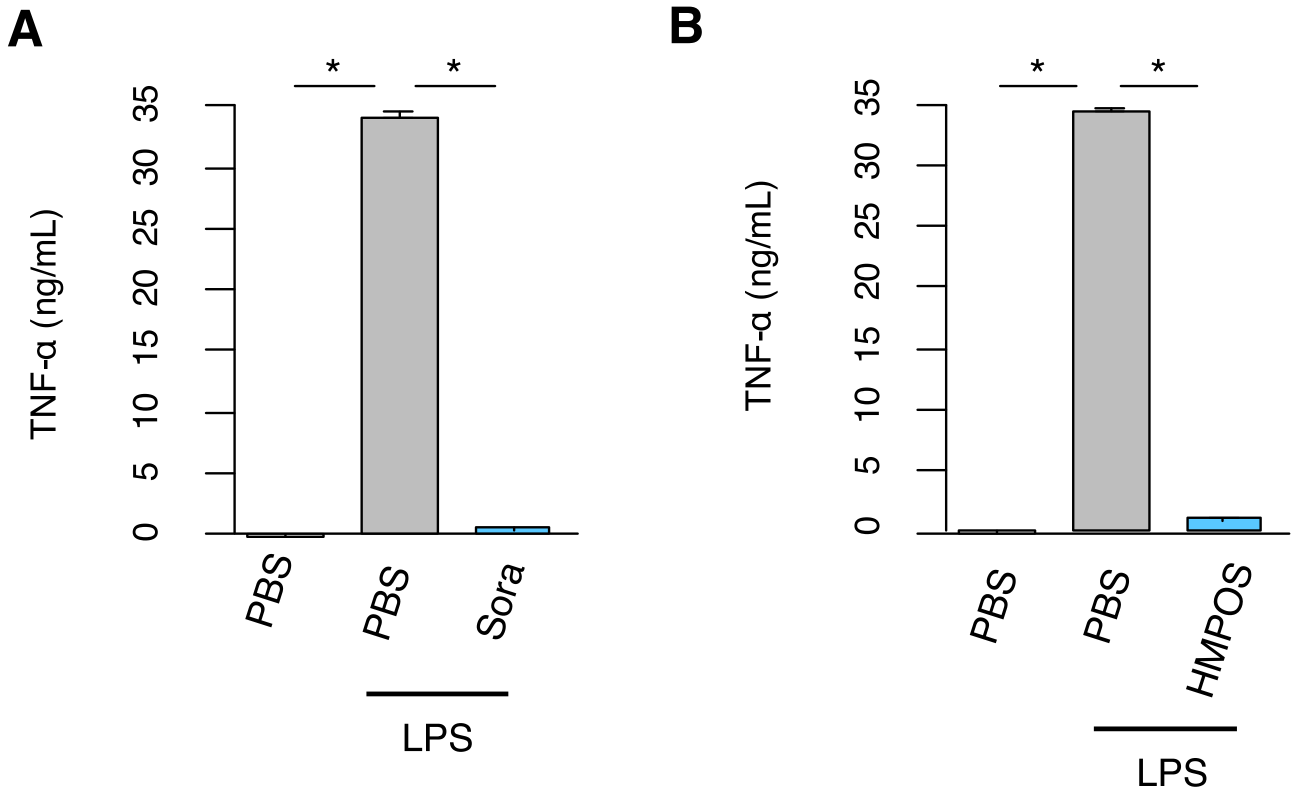

Supplementary Fig.S2

Suppression of LPS-induced TNF-α production by supernatant of necrotic cells. **(A, B)** RAW264.7 cells were stimulated with LPS (10 ng/ml) for 2 h and co-cultured with necrotic supernatants from Sora **(A)** and HMPOS cells **(B)**. The volume of the supernatant is equivalent to 5×10^6 cells. TNF-α production in cell culture medium was then measured by ELISA. Data are presented as mean ± SD. *, $p < 0.05$ compared with the indicated samples.

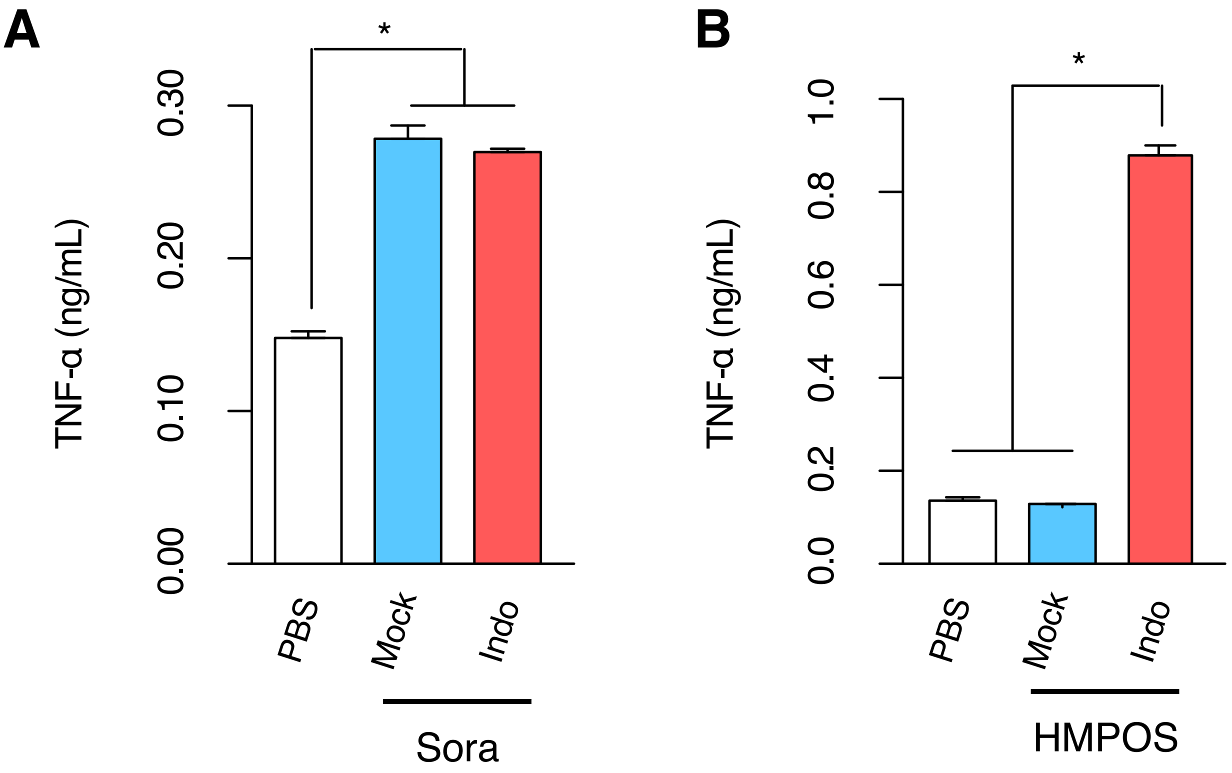

Supplementary Fig.S3

Enhancement of immunostimulatory activity of necrotic cell supernatants by the inhibition of PGE2 synthesis **(A, B)** RAW264.7 cells were treated for 2 h with necrotic supernatant from Mock- or Indo-treated Sora **(A)** and HMPOS **(B)** cells (5×10^6 cells). TNF-a production in cell culture medium was then measured by ELISA. Data are presented as mean ± SD. *, $p < 0.05$ compared with the indicated samples.

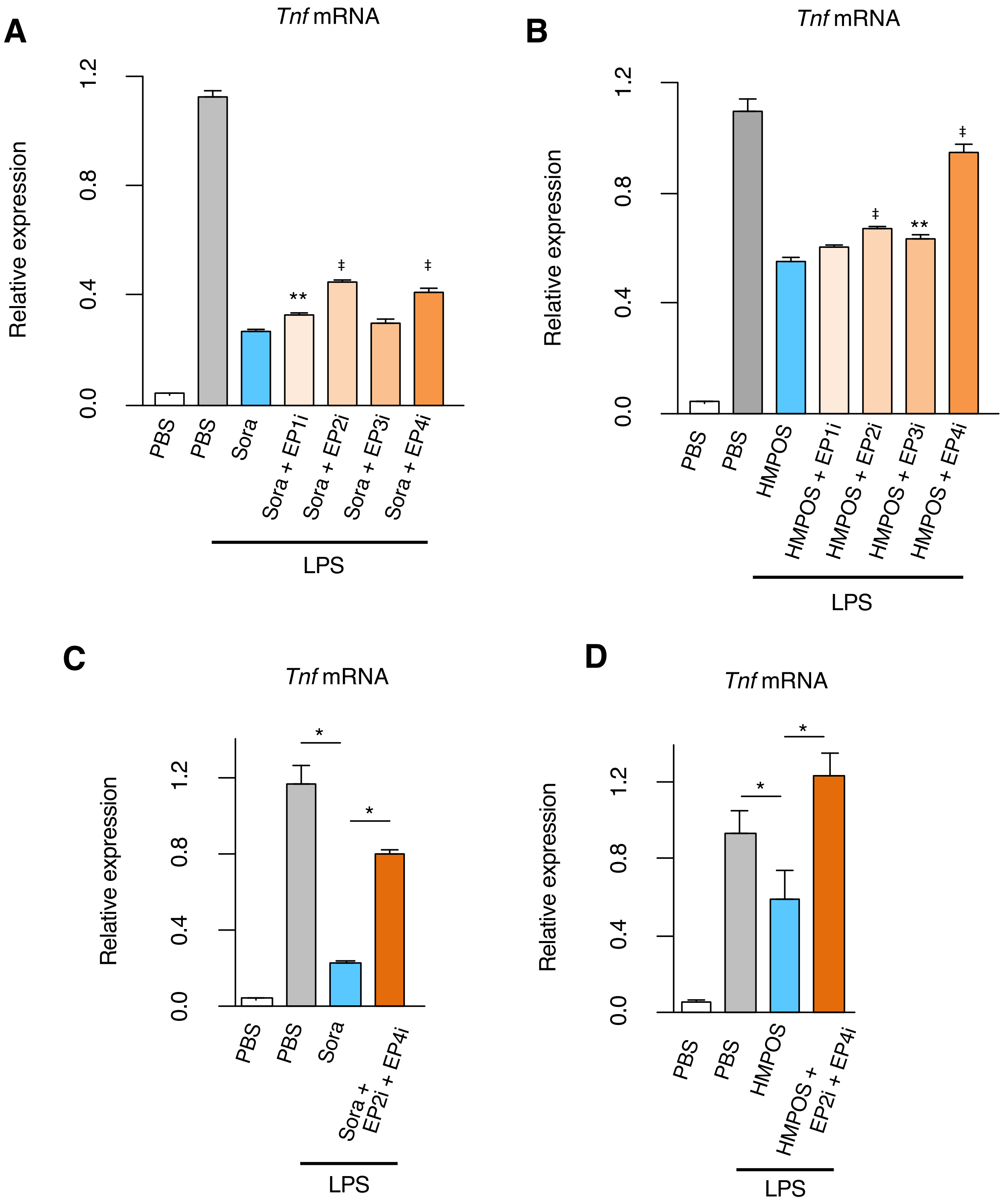

Supplementary Fig.S4

Immunosuppressive effect of dying cell-derived PGE2 via EP receptors. **(A, B)** RAW264.7 cells were stimulated with LPS (10 ng/ml) for 2 h and co-cultured with an increasing volume of necrotic supernatants from Sora **(A)** and HMPOS cells **(B)** in the presence of each EP inhibitor (10 μM). SC51089 (EP1 inhibitor; EP1i), TG4-155 (EP2i), L-798106 (EP3i), and ONO-AE3-208 (EP4i) were used. **(C, D)** RAW264.7 cells were stimulated with LPS (10 ng/ml) for 2 h and co-cultured with necrotic supernatants from Sora **(C)** and HMPOS cells **(D)** in the presence of both EP2i (10 μM) and EP4i (10 μM). The volume of the supernatant is equivalent to 5 × 10^6 cells. *Tnf* mRNA expression levels were examined by RT-qPCR analysis. Data are presented as mean ± SD. *p < 0.05, **p < 0.01, ‡p < 0.001 compared with the indicated samples.

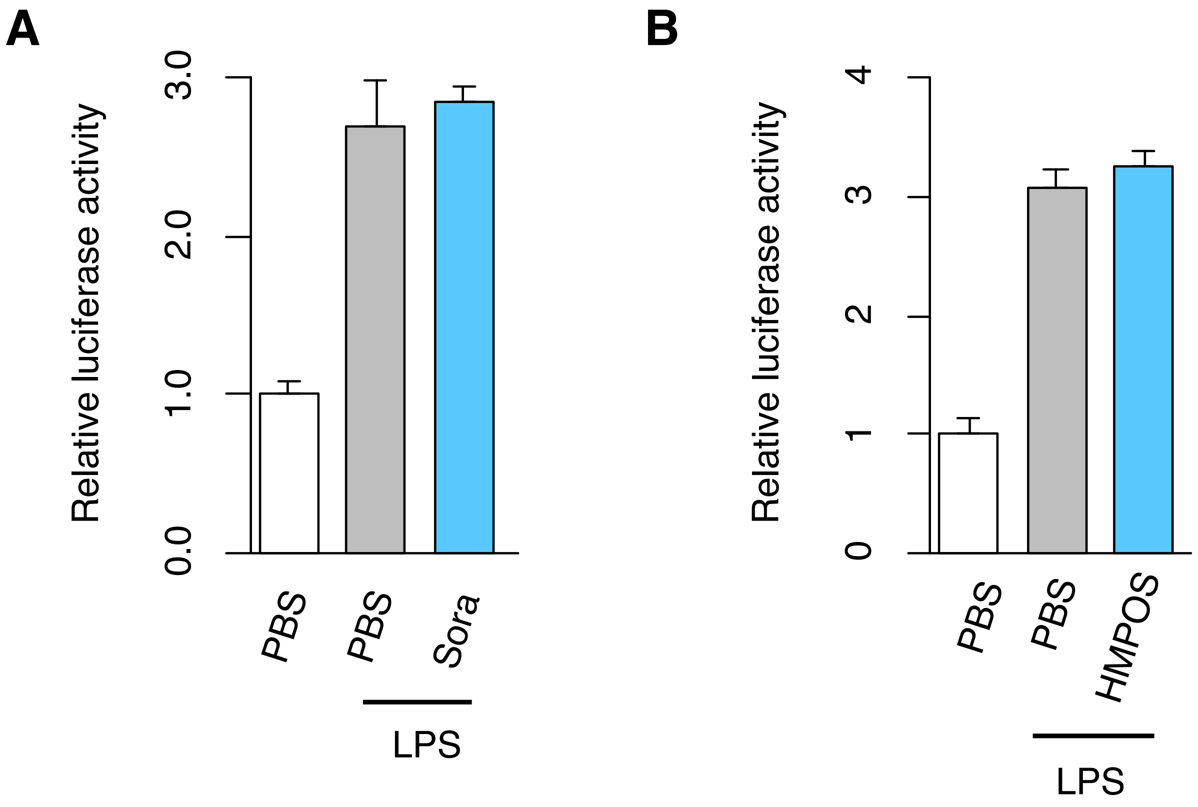

Supplementary Fig.S5

Effect of necrotic cell supernatant on LPS-induced transcriptional activity of NF-κB. **(A, B)** RAW264.7 cells transfected with the NF-κB luciferase reporter gene were stimulated with LPS (10 ng/ml) for 2 h in the presence or absence of necrotic supernatant from Sora (**A**) or HMPOS cells (**B**). Cells were harvested with lysis buffer and luciferase activity was measured. Data are presented as mean ± SD.
